# NELA, P-POSSUM, and Paraspinal Muscle Index in risk stratification of elderly patients undergoing emergency laparotomy

**DOI:** 10.1186/s12877-026-07539-y

**Published:** 2026-05-11

**Authors:** Mehmet Alperen Ugur, Gokalp Okut, Rasit Eren Buyuktoka, Atalay Aktuna, Gulec Mert Dogan, Savas Yakan

**Affiliations:** 1 Department of General Surgery and Organ Transplantation, University of Health Sciences, Izmir City Hospital, Izmir, Türkiye; 2 Department of Gastrointestinal Surgery and Organ Transplantation, University of Health Sciences, Izmir City Hospital, Izmir, Türkiye; 3 Department of Radiology, University of Health Sciences. Izmir City Hospital, Izmir, Türkiye; 4 Department of Public Health, Bandırma Onyedi Eylul University, Bandırma, Balıkesir, Türkiye; 5 Department of General Surgery, University of Health Sciences, Tepecik Education and Research Hospital, Izmir, Türkiye

**Keywords:** Emergency laparotomy, Risk stratification, Paraspinal Muscle Index, Sarcopenia, Prognostic Nutritional Index, Elderly patients

## Abstract

**Background:**

Emergency laparotomy in older adults is associated with substantial morbidity and mortality, necessitating accurate and rapid preoperative risk stratification. Established risk models such as National Emergency Laparotomy Audit (NELA) and the Portsmouth Physiological and Operative Severity Score for the enUmeration of Mortality and Morbidity (P-POSSUM) are widely used, while computed tomography (CT)-derived sarcopenia indices have emerged as potential adjunctive markers of physiological vulnerability. However, the comparative performance of clinical risk scores and paraspinal muscle–based morphometric indices in elderly emergency surgery remains uncertain.

The aim of this study was to evaluate the comparative performance of CT-derived morphometric indices, established risk models (NELA and P-POSSUM), and the Prognostic Nutritional Index (PNI) in predicting postoperative morbidity and in-hospital mortality in elderly patients undergoing emergency laparotomy.

**Methods:**

This prospective, single-center cohort study included patients aged ≥65 years who underwent emergency laparotomy for acute abdomen between 2022 and 2023. Preoperative risk assessment included the NELA, P-POSSUM, and PNI scores. CT-derived sarcopenia was assessed using the Paraspinal Muscle Index (PSMI) and Fat-to-Muscle Ratio (FMR), measured on contrast-enhanced abdominal CT at the L3 level. Postoperative complications were graded according to the Clavien–Dindo classification. Correlation analyses and receiver operating characteristic (ROC) curve analyses were performed to evaluate associations with postoperative morbidity and in-hospital mortality.

**Results:**

A total of 113 patients were included (mean age 74.1 ± 8.1 years; 61.1% male). Major postoperative complications (Clavien–Dindo ≥ 3) and in-hospital mortality were strongly associated with nutritional status. Lower PNI values were significantly associated with major complications, life-threatening complications (Clavien–Dindo ≥ 4), and in-hospital mortality. A PNI cut-off value of approximately 34.5 demonstrated moderate discriminative performance (Area under the curve (AUC) 0.760–0.768), with an AUC of 0.761 (95% CI 0.659–0.851) for mortality prediction.

In contrast, PSMI and FMR showed no significant association with postoperative complication severity (PSMI: *p* = 0.479; FMR: *p* = 0.614) or mortality and did not correlate with clinical risk scores (all *p* > 0.05).

As expected, NELA and P-POSSUM were significantly associated with postoperative outcomes (all p<0.001). However, the addition of PNI did not significantly improve model discrimination (NELA vs. NELA+PNI: ΔAUC=0.001, *p*=0.77; P-POSSUM vs. P-POSSUM+PNI: ΔAUC=0.032, *p*=0.17).

**Conclusions:**

In elderly patients undergoing emergency laparotomy, established clinical risk scores and nutritional status were strongly associated with postoperative outcomes, whereas CT-derived paraspinal muscle quantity indices did not reflect perioperative risk or complication severity. Future predictive models should integrate nutritional indices, muscle quality measures, functional frailty, and acute physiological parameters to improve risk stratification in elderly emergency surgery.

**Supplementary Information:**

The online version contains supplementary material available at 10.1186/s12877-026-07539-y.

## Introduction

Sarcopenia is a clinical condition characterized by an age-related decline in skeletal muscle mass, muscle strength, and/or physical performance, and is closely associated with functional capacity in the geriatric population. Increasing evidence indicates that sarcopenia adversely affects outcomes following trauma and emergency surgery in older patients, contributing to higher morbidity and mortality rates [1]. 

Despite growing interest, there is no universal consensus regarding the optimal method for assessing sarcopenia and frailty. Various approaches have been described, including clinical frailty scales and imaging-based techniques such as ultrasonography, dual-energy X-ray absorptiometry, magnetic resonance imaging, and computed tomography (CT) [2, 3]. In the emergency surgery setting, routine preoperative abdominal CT—already obtained for diagnostic purposes—offers a unique opportunity to incorporate a rapid, reproducible, and objective imaging-based marker of vulnerability or sarcopenia into clinical decision-making, without additional cost or delay.

The psoas major muscle has been widely used as a practical surrogate marker of skeletal muscle mass, with several studies demonstrating its prognostic value across different surgical populations [4]. Psoas-based assessment typically involves measurement of cross-sectional muscle area with normalization for body size; however, considerable methodological heterogeneity exists, including normalization by height, vertebral body area at the level of the third lumbar vertebra (L3), or body surface area [5].

However, reliance on a single muscle group such as the psoas has important limitations, as it may not adequately represent total skeletal muscle mass and can be influenced by local anatomical variability, posture-related loading, and disease-specific changes. In contrast, total skeletal muscle area at the L3 level has been shown to provide a more robust and representative estimate of whole-body muscle mass [5, 6].

Accordingly, alternative muscle groups have been investigated in CT-based morphometric analysis. The paraspinal (erector spinae) muscle group represents a larger and more consistently visualized muscle compartment on routine abdominal CT, with potentially greater anatomical stability and measurement reproducibility. Therefore, in the present study, we utilized the paraspinal muscle group at the L3 level to calculate the Paraspinal Muscle Index (PSMI), particularly considering the need for rapid and pragmatic assessment in the emergency setting, where full skeletal muscle segmentation may not be feasible.

Beyond muscle quantity, nutritional and immunological reserve has emerged as a key determinant of postoperative outcomes in elderly and high-risk surgical populations. The Prognostic Nutritional Index (PNI), derived from serum albumin concentration and peripheral lymphocyte count, reflects both nutritional status and immune competence. PNI has been shown to predict postoperative morbidity and mortality in gastrointestinal and emergency surgical settings, particularly in older patients, and may capture a dimension of physiological vulnerability that complements morphometric sarcopenia measures and traditional risk scores. Importantly, PNI can be readily calculated from routine preoperative laboratory data, making it a practical tool for rapid risk stratification in emergency surgery [7].

Within this framework, the present study aimed to evaluate the relationship between CT-derived body composition indices and a simple laboratory-based nutritional marker with established perioperative risk models in elderly patients undergoing emergency laparotomy, and to explore their potential role in preoperative risk assessment for postoperative morbidity and in-hospital mortality. Specifically, we compared the predictive value of CT-based sarcopenia measures and PNI with established perioperative risk models—the National Emergency Laparotomy Audit (NELA) score and the Portsmouth Physiological and Operative Severity Score for the enUmeration of Mortality and Morbidity (P-POSSUM)—for postoperative morbidity and mortality in patients aged 65 years and older undergoing emergency laparotomy for acute abdomen [8, 9].

In this study, sarcopenia assessment was based on preoperative contrast-enhanced abdominal CT images obtained at the level of the L3 vertebra. PSMI was calculated by dividing the paraspinal muscle area by the square of the patient’s height (cm²/m²). The Fat-to-Muscle Ratio (FMR) was defined as the ratio of subcutaneous adipose tissue area to paraspinal muscle area measured at the same anatomical level. This parameter was calculated to reflect the relative balance between adiposity and skeletal muscle mass, which has been proposed as an additional body composition marker reflecting metabolic reserve and nutritional status in surgical populations [10]. By integrating CT-based morphometric indices with PNI and established clinical risk scores, this study sought to identify rapid, objective markers that could help flag high-risk elderly patients preoperatively and support perioperative decision-making in emergency abdominal surgery.

## Materials and methods

### Study design and patient selection

This study was designed as a prospective, analytical, observational, single-center clinical cohort study. Patients aged 65 years or older who underwent emergency laparotomy with a preliminary diagnosis of acute abdomen at a single tertiary center between 2022 and 2023 were prospectively followed according to a predefined study protocol.

In all patients, NELA and P-POSSUM scores were calculated prospectively in the preoperative period, and postoperative clinical course and outcomes were recorded in a forward-looking manner.

Patients whose postoperative follow-up was continued at another institution; those operated on for acute appendicitis, perforated or necrotic cholecystitis, or blunt or penetrating abdominal trauma (These conditions were excluded because they represent distinct emergency surgical entities with different operative complexity and perioperative risk profiles compared with major emergency laparotomy procedures. Similar exclusion criteria have been applied in previous studies evaluating perioperative risk prediction models and body composition parameters in emergency laparotomy populations [8, 9, 11]). ; patients who did not undergo preoperative intravenous contrast-enhanced abdominal CT; patients whose CT images were unsuitable for evaluation due to technical issues during image acquisition (including motion artifacts, inadequate contrast enhancement, or incomplete imaging coverage); patients for whom PNI, NELA, and/or P-POSSUM scores could not be calculated; and patients who did not provide written informed consent were excluded from the study.

### Imaging protocol and body composition analysis

All patients underwent preoperative intravenous contrast-enhanced, dynamic whole-abdomen CT imaging according to a standardized protocol. CT images were acquired with a slice thickness of 3 mm.

Body composition analysis of CT images was performed after completion of the study by two independent radiology specialists. The radiologists were blinded to patients’ clinical data, surgical course, and study hypothesis and conducted the assessments in accordance with a blinded evaluation protocol. Final measurements were derived from the mean of the two independent radiological assessments.

Muscle and subcutaneous adipose tissue measurements were performed on a single axial slice at the level of the L3 vertebra. Paraspinal muscle area and subcutaneous adipose tissue area measured at this level were used to calculate body composition parameters.

To assess measurement reliability prior to the study, 20 pilot CT images were independently analyzed by both radiologists without mutual awareness. Interobserver agreement was found to be 90%, which was considered an acceptable level of measurement reliability.

Based on these measurements:

PSMI was defined as the paraspinal muscle area divided by the square of the patient’s height (cm²/m²). The paraspinal muscle area measured at the L3 level included the erector spinae muscle group (iliocostalis, longissimus, and spinalis muscles), which are consistently visualized on routine abdominal CT scans and commonly used in CT-based morphometric body composition analysis.

FMR was defined as the ratio of the subcutaneous adipose tissue area to the paraspinal muscle area measured at the same level.

Image segmentation and area measurements were performed using dedicated radiological analysis software (Horos software, v3.3.5, Annapolis, MD, USA).

### Assessment of nutritional status

Patients’ nutritional status was evaluated using the Prognostic Nutritional Index (PNI). PNI was calculated based on preoperative serum albumin level (g/L) and total lymphocyte count (×10⁹/L) using the following formula:

PNI = (10 × serum albumin) + (0.005 × total lymphocyte count).

PNI was included in the analyses as a marker reflecting patients’ nutritional and immunological reserve, and its associations with perioperative risk scores and clinical outcomes were evaluated.

### Clinical outcomes

Postoperative surgical, cardiac, and/or respiratory complications were recorded. In addition, consultations planned and performed by the treating clinicians, clinical follow-up processes, non-invasive treatments, reoperations, intensive care unit admissions, need for endotracheal intubation, discharge timing, and, in patients who died, time to death were documented.

All postoperative complications were classified according to the Clavien–Dindo classification.

### Ethical approval and informed consent

This study was conducted in accordance with the principles of the Declaration of Helsinki. The study protocol was approved by the Institutional Clinical Research Ethics Committee of University of Health Sciences Bozyaka Training and Research Hospital (approval number and date: [168/2022]). Written informed consent was obtained from all participants prior to enrollment, or from legally authorized representatives in cases where patients lacked decision-making capacity. Patients who declined participation or did not provide informed consent were not included in the study.

### Statistical analysis

The distribution characteristics of FMR and PSMI were assessed using histogram plots, Kolmogorov–Smirnov, and Shapiro–Wilk tests. For variables showing normal distribution, comparisons between two independent groups were performed using the Student’s t-test, and comparisons among three or more groups were conducted using one-way analysis of variance (ANOVA).

When normal distribution assumptions were not met, the Mann–Whitney U test was used for comparisons between two groups, and the Kruskal–Wallis H test was applied for comparisons among three or more groups. Following Kruskal–Wallis analysis, pairwise comparisons were conducted using the Mann–Whitney U test with Bonferroni correction.

Associations among PNI, NELA, P-POSSUM scores, and Clavien–Dindo classification were evaluated using correlation analyses and receiver operating characteristic (ROC) curve analyses. In addition to PNI, CT-derived morphometric indices (PSMI and FMR) were also evaluated for their associations with postoperative outcomes and complication severity using group comparisons and correlation analyses. A p value < 0.05 was considered statistically significant. The optimal cut-off value for PNI was determined using ROC curve analysis based on the Youden index.

To explore whether nutritional status could improve the predictive performance of existing risk scores, combined predictive models were constructed. Specifically, PNI + NELA and PNI + P-POSSUM models were generated using binary logistic regression analysis, in which in-hospital mortality was used as the dependent variable and the respective predictors were entered simultaneously into the model. Predicted probabilities obtained from these regression models were then used to generate ROC curves and calculate the corresponding Area under the curve (AUC).

All statistical analyses were performed using IBM SPSS Statistics for Windows, Version 24.0 (IBM Corp., Armonk, NY, USA).

### Sample size considerations

No formal a priori sample size or power calculation was performed. The study cohort consisted of all consecutive eligible patients aged 65 years and older who underwent emergency laparotomy at our institution during the predefined study period (2022–2023). Therefore, the present study should be interpreted as a prospective exploratory cohort analysis.

## Results

**Fig. 1:**
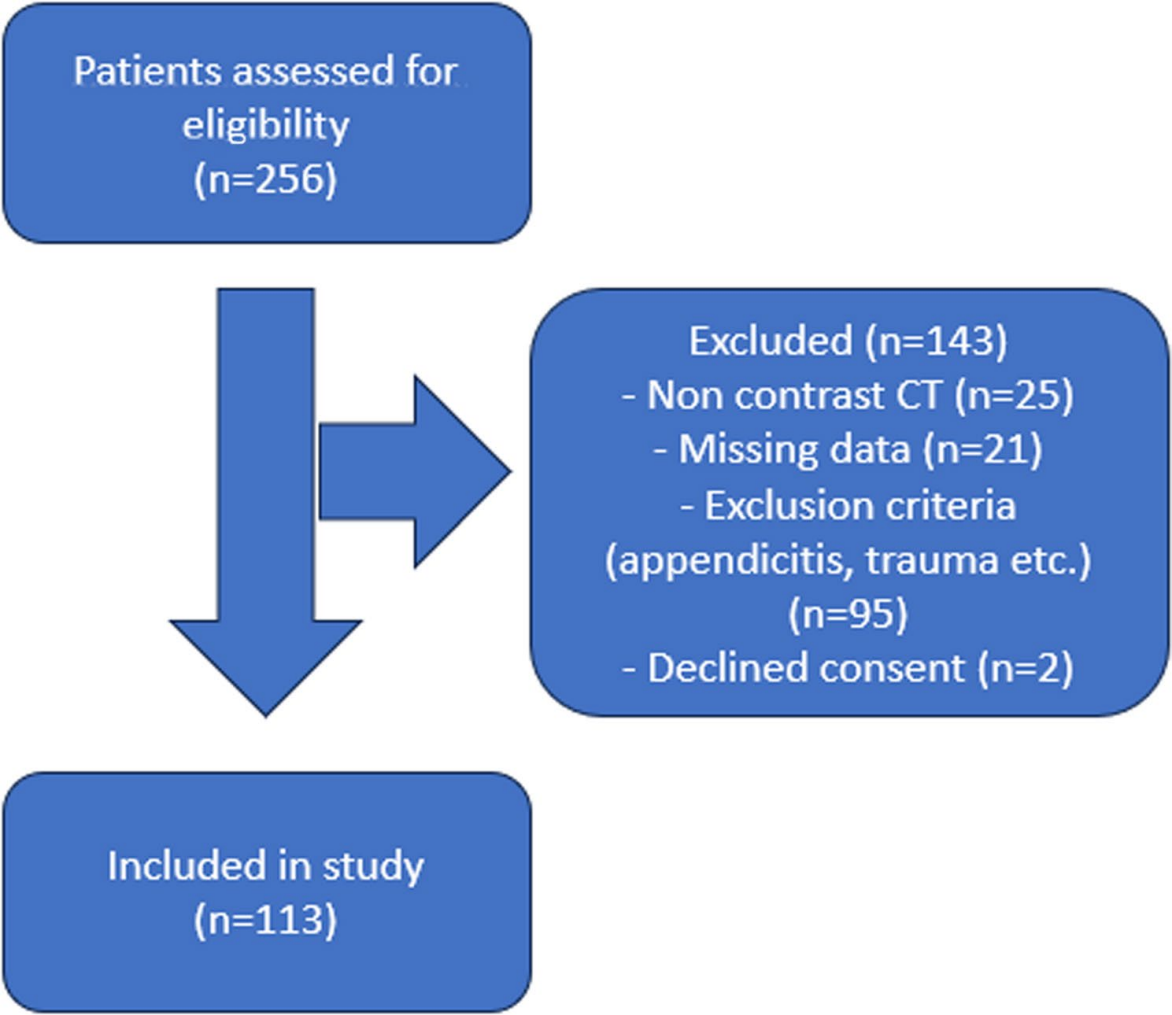
Flow diagram of patient selection and inclusion process. A total of 256 patients aged ≥ 65 years who underwent emergency laparotomy were assessed for eligibility. Patients were excluded due to absence of contrast-enhanced CT imaging, missing data, not meeting inclusion criteria (appendicitis, cholecystitis, or trauma), or lack of informed consent. The final study cohort consisted of 113 patients

A flow diagram illustrating patient selection and exclusion process is presented in Fig. 1.

A total of 113 patients were included in the study. Of these, 69 patients (61.1%) were male and 44 (38.9%) were female. The mean age of the cohort was 74.1 ± 8.1 years (median, 73 years). Sixty-four patients (56.6%) were younger than 75 years, whereas 49 patients (43.4%) were aged 75 years or older.

The most common surgical indications were incarcerated hernias (21.2%, *n* = 24), peptic ulcer perforation (17.7%, *n* = 20), and colonic tumor–related obstruction (11.5%, *n* = 13). Of the 113 patients, 33 (29.2%) had malignant disease, whereas 80 (70.8%) were diagnosed with benign pathology. Malignancy was more frequent in patients aged ≥ 75 years compared with younger patients (40.8% vs. 20.3%, *p* = 0.017), whereas no significant difference was observed by sex (33.3% vs. 22.7%, *p* = 0.22).

Median PNI values did not differ significantly between malignant and benign cases (37.05 [IQR 29.00–40.30] vs. 37.60 [30.56–42.84], *p* = 0.166), nor did NELA scores (16.91% [10.84–27.54] vs. 13.88% [6.30–25.52], *p* = 0.116). In contrast, P-POSSUM–predicted mortality (33.20% [23.00–42.60] vs. 22.35% [9.82–41.60], *p* = 0.012) and morbidity (84.80% [75.40–90.40] vs. 74.45% [47.60–89.80], *p* = 0.008) were significantly higher in malignant disease. Actual in-hospital mortality did not differ between malignant and benign groups (30.3% vs. 20.0%, *p* = 0.325).

Anthropometric and CT-based body composition measurements are summarized in Table 1. In this cohort, the mean paraspinal muscle area was 37.562 ± 9.536 cm², and the mean subcutaneous adipose tissue area was 174.150 ± 116.797 cm². The mean PSMI was calculated as 13.332 ± 3.07 cm²/m², while the mean FMR was 5.20 ± 4.38.

**Table 1:** Anthropometric characteristics and CT-derived body composition parameters of the study population

Variable	Mean ± SD	Min-Max
Height (cm)	167.5 ± 8.9	150–190
Weight (kg)	72.0 ± 13.5	40–110
Body Surface Area (cm²)	281.3 ± 29.6	225–361
Body Mass Index (kg/m²)	25.7 ± 4.9	14.7–44.4
Waist Circumference (cm)	98.22 ± 14.72	58.59–142.83
Psoas Muscle Area (cm²)	142.64 ± 53.73	14.72–292.82
Paraspinal Muscle Area (cm²)	37.56 ± 9.54	11.40–60.17
Subcutaneous Fat Area (cm²)	174.15 ± 116.80	77.51–605.4
PSMI (cm²/m²)	13.33 ± 3.07	5.07–20.04
FMR	5.20 ± 4.38	0.27–25.86

According to the Clavien–Dindo classification, postoperative complications were observed as follows: Grade II in 22 patients (19.8%), Grade III in 12 patients (10.8%), and Grade IV in 12 patients (10.8%).

Because CT-derived morphometric indices (PSMI and FMR) did not demonstrate significant associations with postoperative complications or mortality in the preliminary analyses, ROC analysis was not performed for these variables.

ROC analysis was performed to evaluate the ability of the Prognostic Nutritional Index (PNI) to predict postoperative adverse outcomes. A PNI cut-off value of 34.5 was identified as the optimal threshold for predicting major complications (Clavien–Dindo ≥ 3), life-threatening complications (Clavien–Dindo ≥ 4), and in-hospital mortality.

For major complications (Clavien–Dindo ≥ 3), the AUC was 0.760 (95% CI 0.668–0.852), with a sensitivity of 63.3% (95% CI 48.3–76.6) and specificity of 73.4% (95% CI 60.9–83.7). The positive likelihood ratio was 2.38 (95% CI 1.52–3.71) and the negative likelihood ratio was 0.50 (95% CI 0.34–0.73).

For life-threatening complications (Clavien–Dindo ≥ 4), the AUC was 0.768 (95% CI 0.676–0.860), with a sensitivity of 64.9% (95% CI 47.5–79.8) and specificity of 68.4% (95% CI 56.7–78.6). The positive likelihood ratio was 2.05 (95% CI 1.40–3.01) and the negative likelihood ratio was 0.51 (95% CI 0.32–0.81).

For in-hospital mortality, the AUC was 0.761 (95% CI 0.659–0.851), with a sensitivity of 76.9% (95% CI 56.4–91.0) and specificity of 67.8% (95% CI 56.9–77.4). The positive likelihood ratio was 2.39 (95% CI 1.66–3.44) and the negative likelihood ratio was 0.34 (95% CI 0.17–0.69).

**Fig. 2:**
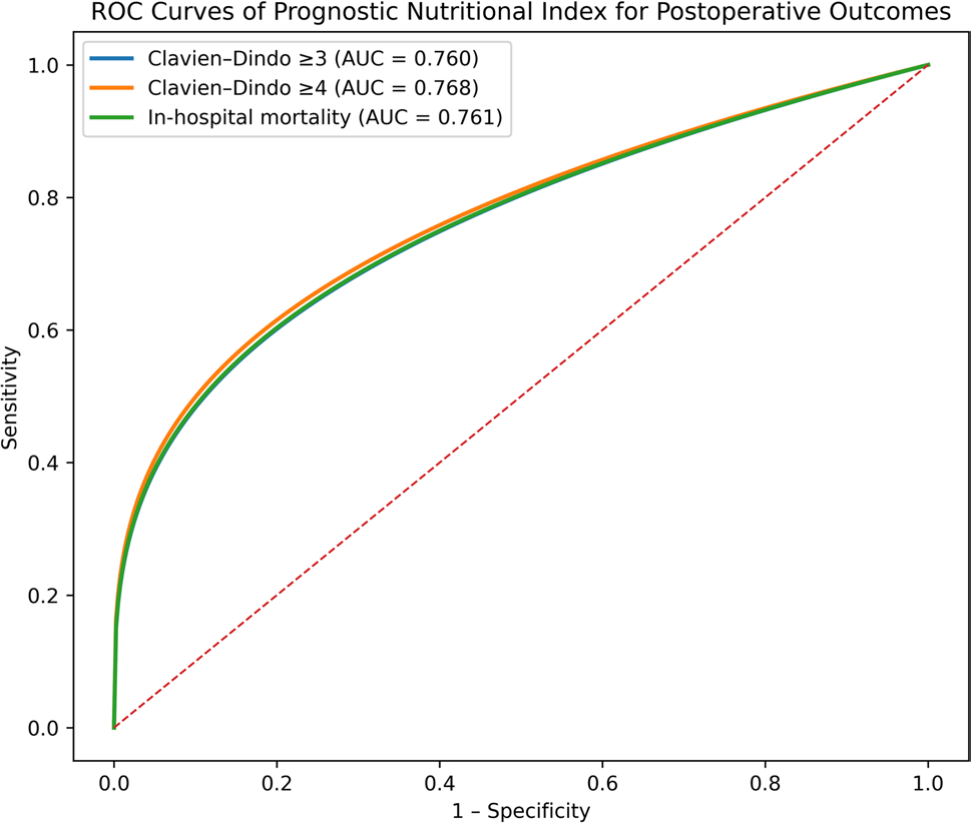
Receiver operating characteristic (ROC) curves of the Prognostic Nutritional Index for predicting postoperative outcomes. ROC analysis identified a PNI cut-off value of 34.5 for predicting major complications (Clavien–Dindo ≥ 3), life-threatening complications (Clavien–Dindo ≥ 4), and in-hospital mortality. The discriminative ability of PNI was moderate across these outcomes, with AUC values of 0.760, 0.768, and 0.761, respectively

The ROC curves illustrating the diagnostic performance of PNI for these outcomes are presented in Fig. 2.

**Fig. 3:**
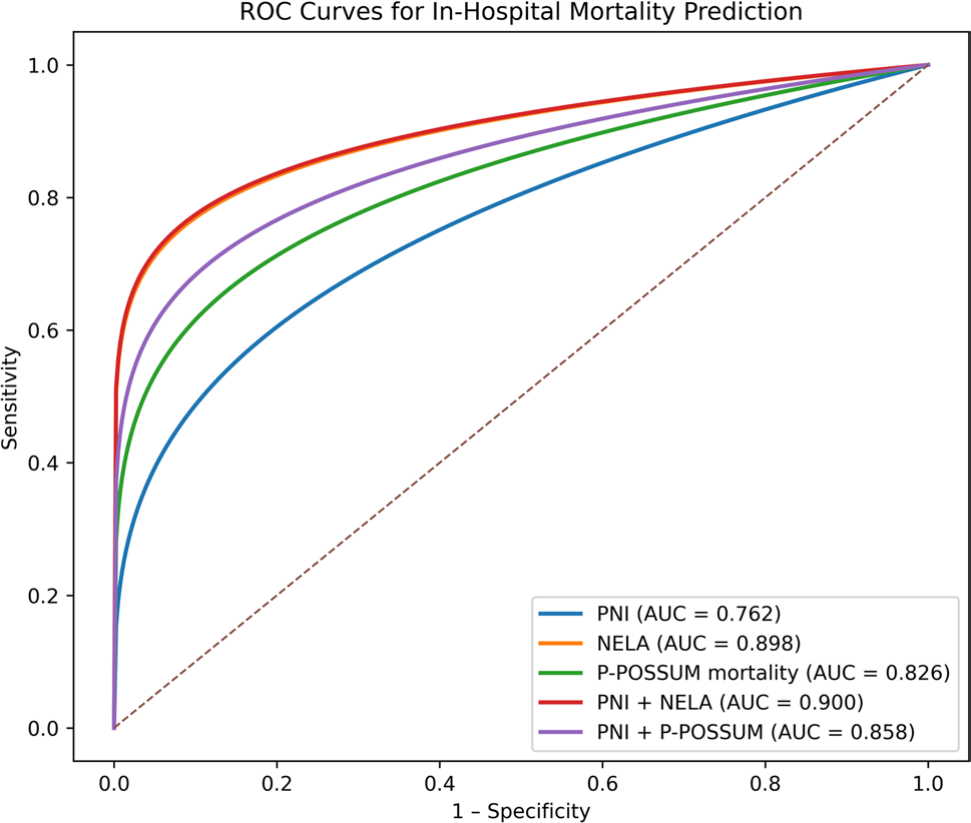
Receiver operating characteristic (ROC) curves for prediction of in-hospital mortality in elderly patients undergoing emergency laparotomy. The NELA score demonstrated the highest discriminative performance (AUC = 0.898), followed by P-POSSUM mortality (AUC = 0.826) and the Prognostic Nutritional Index (PNI) (AUC = 0.762). The combined PNI+NELA model yielded an AUC of 0.900, which was not significantly different from NELA alone (ΔAUC = 0.001, *p* = 0.77). Similarly, the addition of PNI to P-POSSUM increased the AUC to 0.858 without reaching statistical significance (ΔAUC = 0.032, *p* = 0.17)

To compare the prognostic performance of different risk models for in-hospital mortality, ROC curves were generated for PNI, NELA, and P-POSSUM mortality scores. The NELA score demonstrated the highest discriminative performance (AUC 0.898), followed by P-POSSUM mortality (AUC 0.826) and PNI (AUC 0.762). When PNI was combined with NELA in a logistic model, the AUC increased slightly to 0.900, although this improvement was not statistically significant compared with NELA alone (ΔAUC = 0.001, *p* = 0.77). Similarly, adding PNI to P-POSSUM increased the AUC to 0.858, but this difference also did not reach statistical significance (ΔAUC = 0.032, *p* = 0.17). The comparative ROC curves illustrating the diagnostic performance of these models are shown in Fig. 3.

When postoperative variables were compared according to FMR, patients requiring additional postoperative imaging had significantly lower FMR values (*p* = 0.014). No other postoperative variables showed a significant association with FMR and PSMI (Tables 2 and 3).

**Table 2:** Statistical comparison of fat-to-muscle ratio (FMR) according to postoperative clinical variables

Variable	Group	Mean ± SD	Range (Min–Max)	*p*
Surgical site infection	No	5.41 ± 4.48	0.27–25.87	0.122ᵃ
	Yes	3.69 ± 3.35	0.46–12.51	
Reoperation	No	5.30 ± 4.57	0.27–25.87	0.923ᵃ
	Yes	4.62 ± 3.13	0.46–12.51	
Stoma requirement	No	5.28 ± 4.59	0.27–25.87	0.368ᵇ
	Ileostomy	4.30 ± 4.32	1.11–18.22	
	Colostomy	5.63 ± 3.33	1.08–13.19	
Blood transfusion	No	5.66 ± 4.63	0.59–25.87	0.098ᵃ
	Yes	4.27 ± 3.73	0.27–18.22	
Antibiotic use	No	5.76 ± 4.81	0.69–25.87	0.254ᵃ
	Yes	4.77 ± 4.00	0.27–18.59	
TPN	No	5.50 ± 4.54	0.46–25.87	0.052ᵃ
	Yes	3.35 ± 2.71	0.27–7.47	
Oral intake status	R0	5.50 ± 4.11	0.69–18.22	0.471ᵃ
	R1–2–3	5.14 ± 4.46	0.27–25.87	
Respiratory pathology	No	5.53 ± 4.55	0.69–25.87	0.305ᵃ
	Yes	4.78 ± 4.17	0.27–18.59	
Cardiac pathology	No	5.41 ± 4.42	0.43–25.87	0.309ᵃ
	Yes	4.75 ± 4.33	0.27–18.59	
Inotropic support	No	5.03 ± 4.22	0.27–25.87	0.413ᵃ
	Yes	6.19 ± 5.26	1.08–18.59	
Surgical pathology (malignancy)	No	5.31 ± 4.57	0.27–25.87	0.792ᵃ
	Yes	4.75 ± 3.58	0.46–13.19	
Additional postoperative imaging	No	5.71 ± 4.62	0.27–25.87	**0.014ᵃ**
	Yes	3.63 ± 3.15	0.43–12.51	
Discharge status	No	5.12 ± 4.33	0.43–25.87	0.736ᵃ
	Yes	5.47 ± 4.65	0.27–18.59	

*TPN* Total parenteral nutrition

^a^Mann-Whitney u testi, ^b^Kruskal-Wallis testi

**Table 3:** Statistical comparison of Paraspinal Muscle Index (PSMI) according to postoperative clinical variables

Variable	Group	Mean ± SD	Range (Min–Max)	*p*
Surgical site infection	No	13.22 ± 3.08	5.07–20.04	0.366ᵃ
	Yes	14.16 ± 2.95	8.28–19.65	
Stoma requirement	No	13.22 ± 3.00	5.07–20.04	0.647ᵇ
	Ileostomy	13.42 ± 3.43	6.39–19.65	
	Colostomy	13.88 ± 3.37	5.33–19.29	
Erythrocyte replacement therapy	No	13.28 ± 2.88	5.07–20.04	0.659ᵃ
	Yes	13.45 ± 3.47	6.39–19.65	
Antibiotic use	No	13.13 ± 2.95	5.33–20.04	0.535ᶜ
	Yes	13.49 ± 3.18	5.07–19.65	
TPN	No	13.37 ± 3.02	5.07–20.04	0.730ᶜ
	Yes	13.08 ± 3.45	6.80–19.29	
Oral intake status	R0	13.49 ± 3.33	6.39–19.29	0.808ᵃ
	R1–2–3	13.33 ± 3.03	5.07–20.04	
Respiratory pathology	No	13.57 ± 2.84	5.33–20.04	0.344ᶜ
	Yes	13.01 ± 3.35	5.07–19.29	
Cardiac pathology	No	13.34 ± 2.94	5.33–20.04	0.944ᵃ
	Yes	13.31 ± 3.39	5.07–19.29	
Inotropic support	No	13.42 ± 2.84	5.33–20.04	0.673ᵃ
	Yes	12.87 ± 4.19	5.07–19.29	
Surgical pathology (malignancy)	No	13.23 ± 3.01	5.07–20.04	0.454ᶜ
	Yes	13.79 ± 3.35	5.33–19.65	
Reoperation	No	13.17 ± 3.02	5.07–20.04	0.165ᶜ
	Yes	14.32 ± 3.30	5.33–19.65	
Additional postoperative imaging	No	13.27 ± 3.10	5.07–20.04	0.697ᶜ
	Yes	13.53 ± 3.03	6.80–19.29	
In-hospital mortality	No	12.84 ± 3.61	5.07–19.29	0.446ᵃ
	Yes	13.48 ± 2.89	5.33–20.04	

*TPN* Total parenteral nutrition

^a^Mann-Whitney u testi, ^b^Kruskal-Wallis testi, ^c^Bonferroni-adjusted Mann–Whitney U test

As complication severity increased according to the Clavien–Dindo classification, both NELA and P-POSSUM scores rose significantly. For NELA, scores increased from a mean of 10.2% in Clavien-Dindo Score (CDS) 1 to 41.2% in CDS 5 (*p* < 0.001). Similarly, P-POSSUM–predicted mortality increased from a mean of 14.82% in CDS 1 to 53.43% in CDS 5 (*p* < 0.001), and P-POSSUM–predicted morbidity increased from 53.78% to 90.93% across the same categories (*p* < 0.001) (Table 4).

**Table 4:** Comparison of risk scores and body composition indices according to Clavien–Dindo classification

Variable	CDS 1	CDS 2	CDS 3	CDS 4	CDS 5	*p* value
NELA score (%)	10.2 ± 4.6	15.8 ± 6.9	26.9 ± 9.3	34.7 ± 10.8	41.2 ± 11.6	< 0.001
P-POSSUM mortality (%)	14.8 ± 6.3	24.7 ± 9.7	38.9 ± 12.6	49.4 ± 14.9	53.4 ± 15.7	< 0.001
P-POSSUM morbidity (%)	53.8 ± 18.3	71.4 ± 15.9	85.0 ± 10.7	89.3 ± 8.6	90.9 ± 7.8	< 0.001
PSMI (cm²/m²)	13.5 ± 3.1	13.4 ± 3.1	13.4 ± 3.4	13.2 ± 3.3	13.0 ± 3.7	0.479
FMR	5.31 ± 4.41	5.24 ± 4.36	5.02 ± 4.22	4.98 ± 4.11	4.87 ± 4.07	0.614

Kruskal–Wallis test

*CDS* Clavien–Dindo classification, *PSMI* Paraspinal Muscle Index, *FMR* Fat-to-Muscle Ratio

In contrast, neither PSMI nor FMR differed significantly across Clavien–Dindo groups (PSMI *p* = 0.479; FMR *p* = 0.614), indicating that body composition indices did not parallel increasing complication severity in this cohort (Table 4).

Correlation analysis demonstrated strong positive correlations between NELA and P-POSSUM–predicted mortality (*r* = 0.774; *p* < 0.001) and morbidity (*r* = 0.771; *p* < 0.001). NELA also correlated significantly with Clavien–Dindo classification (*r* = 0.637; *p* < 0.001). Significant correlations were likewise observed between Clavien–Dindo and P-POSSUM–predicted mortality (*r* = 0.685; *p* < 0.001) and morbidity (*r* = 0.676; *p* < 0.001) (Table 5).

**Table 5:** Correlation analysis between risk scores, body composition indices, and complication severity

Variable	NELA	*P*-POSSUM Mortality	*P*-POSSUM Morbidity	PSMI	FMR	CDS
NELA	1	0.774***	0.771***	-0.124	-0.127	0.637***
P-POSSUM Mortality		1	0.995***	-0.088	-0.162	0.685***
P-POSSUM Morbidity			1	-0.084	-0.161	0.676***
PSMI				1	-0.287**	-0.013
FMR					1	0.025

Spearman correlation coefficients. **p < 0.01, ***p < 0.001

*CDS* Clavien–Dindo classification, *PSMI* Paraspinal Muscle Index, *FMR* Fat-to-Muscle Ratio

Overall, these findings demonstrate that NELA and P-POSSUM scores are strongly correlated with each other and align closely with postoperative complication severity, whereas CT-derived body composition indices (PSMI and FMR) did not show meaningful correlations with NELA, P-POSSUM, or Clavien–Dindo classification in this cohort (Tables 4 and 5).

## Discussion

Emergency abdominal surgery in older adults represents one of the most challenging clinical scenarios in modern surgical practice, owing to the combined effects of advanced age, reduced physiological reserve, and acute pathological insults. In this setting, accurate preoperative risk stratification is essential to guide decision-making, allocate perioperative resources, and optimize postoperative management. Established risk models such as the NELA score and P-POSSUM remain widely used tools for predicting outcomes following emergency laparotomy and have demonstrated robust discrimination for short-term morbidity and mortality despite calibration variability across healthcare systems [8, 12].

Parallel to the development of clinical risk calculators, CT-derived body composition analysis has gained increasing attention as an objective method for quantifying physiological vulnerability. Radiological sarcopenia—typically assessed at the L3 vertebral level—has been associated with adverse postoperative outcomes in emergency laparotomy populations. A recent systematic review and meta-analysis including 3,492 patients demonstrated that sarcopenia was associated with increased short-term mortality and prolonged hospital stay, suggesting that muscle depletion reflects a dimension of physiological reserve not fully captured by conventional risk models [13]. Furthermore, several cohort studies have suggested that radiological sarcopenia may complement traditional frailty assessments in emergency surgical settings and may improve risk stratification when combined with basic clinical variables [14–17].

Beyond mortality, contemporary cohort studies suggest that radiological sarcopenia may complement or, in selected contexts, even outperform traditional frailty measures in emergency surgical populations. Hajibandeh et al. reported sarcopenia to be a stronger predictor of postoperative mortality than the Clinical Frailty Scale in emergency laparotomy, highlighting the potential value of objective morphometric indices when functional frailty assessment is impractical in urgent clinical settings [14]. Similarly, simplified predictive frameworks combining sarcopenia with basic clinical variables such as American Society of Anesthesiologists (ASA) status have been proposed to enhance risk stratification in emergency laparotomy pathways [15].

The relationship between radiological sarcopenia and established perioperative risk scores has also been directly examined. In the “sarcopenia made simple” study, Ming et al. demonstrated weak but statistically significant correlations between radiological sarcopenia and both NELA and P-POSSUM, suggesting partial overlap between morphometric muscle depletion and physiological or operative severity. At the same time, these findings underscored that sarcopenia indices and risk calculators interrogate distinct dimensions of perioperative risk [16]. This observation aligns with the broader conceptual framework in which sarcopenia represents a morphometric component of frailty that is largely orthogonal to the acute physiological derangements captured by perioperative risk models [17].

In contrast to these reports, our prospective cohort demonstrated no significant association between CT-derived morphometric indices—PSMI and FMR—and NELA, P-POSSUM, or postoperative complication severity as defined by the Clavien–Dindo classification. This absence of correlation was consistent across both group-based comparisons and correlation analyses. In contrast, NELA and P-POSSUM behaved as expected, demonstrating strong mutual correlations and close alignment with increasing Clavien–Dindo severity. These findings indicate that, in our specific population of elderly emergency laparotomy patients, CT-based quantity-derived morphometric indices did not parallel either established clinical risk scores or short-term postoperative outcomes.

First, the use of regional muscle surrogates such as the paraspinal or psoas muscles may limit construct validity when attempting to estimate global skeletal muscle mass. Although psoas muscle area has frequently been used as a surrogate marker of whole-body muscle mass in CT-based sarcopenia studies, subsequent imaging research has demonstrated that measurements derived from a single muscle group may not fully represent total skeletal muscle volume. Studies comparing regional muscle measurements with comprehensive L3 skeletal muscle segmentation have shown that total skeletal muscle area at the L3 level correlates more strongly with whole-body muscle mass than isolated psoas or paraspinal measurements. In addition, individual muscle groups may be influenced by local anatomical variability, posture-related loading patterns, and disease-specific muscle remodeling, which can introduce measurement variability and reduce their ability to reflect systemic muscle depletion in heterogeneous emergency surgical populations [6, 13, 16]. Second, although CT imaging in our study was obtained using a standardized institutional protocol, emergency CT acquisition may still be subject to variability in clinical practice, including differences in contrast phase, acquisition timing, slice thickness, and reconstruction algorithms. Such variability has been reported in the literature and may influence the accuracy of muscle and subcutaneous adipose tissue segmentation in CT-based body composition analysis [17].

Third, acute physiological perturbations commonly encountered in emergency surgery—including sepsis, systemic inflammation, capillary leak, aggressive fluid resuscitation, and preoperative rehydration—may transiently increase muscle cross-sectional area through interstitial edema. Such effects can mask underlying chronic muscle depletion, thereby reducing the ability of CT-based muscle area measurements to reflect baseline physiological reserve and weakening their correlation with short-term outcomes [13]. Furthermore, our analysis did not incorporate muscle quality metrics such as myosteatosis. Emerging evidence suggests that muscle attenuation may be an independent—and in some settings superior—predictor of adverse outcomes compared with muscle quantity alone in emergency laparotomy cohorts, potentially explaining the absence of association observed in our study [11, 18].

In contrast to CT-derived morphometric indices, nutritional and immunological reserve—assessed using the PNI—demonstrated clinically meaningful associations with adverse outcomes in our cohort. Although median PNI values did not differ significantly between malignant and benign disease, lower PNI values were significantly associated with major postoperative complications (Clavien–Dindo ≥ 3), life-threatening complications (Clavien–Dindo ≥ 4), and in-hospital mortality. Receiver operating characteristic analysis identified a consistent PNI cut-off of approximately 34.5 across these outcomes, with moderate-to-good discriminative performance. These findings support the concept that PNI captures a dimension of vulnerability related to chronic nutritional and immunological status that is not adequately reflected by morphometric muscle quantity alone.

The biological plausibility of PNI as a prognostic marker is well established. Originally described by Onodera et al. as a composite index incorporating serum albumin and peripheral lymphocyte count, PNI reflects both protein reserves and immune competence—two key determinants of postoperative recovery and resilience to surgical stress [19]. In gastrointestinal and emergency surgery, low PNI has consistently been associated with increased postoperative morbidity and mortality, particularly in older and high-risk patients, underscoring its relevance in acute surgical settings.

Importantly, while PNI showed significant associations with postoperative complications and mortality, its integration into existing risk models yielded nuanced results. PNI alone demonstrated moderate discriminative ability for in-hospital mortality; however, adding PNI to NELA did not significantly improve the area under the curve, likely reflecting the already high discriminatory power of NELA for acute physiological risk. Similarly, the addition of PNI to P-POSSUM resulted in a numerical increase in AUC that did not reach statistical significance. These findings suggest that PNI provides complementary biological information but may not substantially enhance discrimination when combined with comprehensive physiology-based models, particularly in cohorts with limited sample size and high baseline risk.

Several limitations of this study should be acknowledged. First, the single-center design and relatively modest sample size may limit the generalizability of our findings. Second, the observational nature of the study precludes causal inference. Third, functional frailty measures, such as gait speed or clinical frailty scales, were not incorporated, preventing direct comparison between functional and morphometric vulnerability assessments. Fourth, sarcopenia assessment relied on a regional CT-based muscle quantity measure (paraspinal muscle area) rather than global skeletal muscle segmentation or muscle quality metrics such as myosteatosis, which may provide additional prognostic information. Finally, variability inherent to emergency CT acquisition may have influenced morphometric measurements. In addition, because no formal a priori sample size calculation was performed, the study may have been underpowered to detect small-to-moderate associations, particularly for negative findings involving CT-derived morphometric indices and combined predictive models. Notwithstanding these limitations, the prospective design, standardized imaging protocol, blinded radiological assessment, and comprehensive evaluation of clinical outcomes represent important strengths of the study.

Taken together, our findings underscore that the prognostic value of CT-derived muscle quantity measures in elderly emergency laparotomy patients may be highly context- and methodology-dependent. While established risk scores and nutritional indices demonstrated clear associations with postoperative outcomes, PSMI and FMR alone did not reliably reflect perioperative risk in this vulnerable population. These results highlight the importance of nutritional status and validated clinical risk models in preoperative assessment and caution against overreliance on isolated CT muscle measures. Future research should focus on integrated predictive frameworks that combine nutritional indices, muscle quality metrics, functional frailty assessments, and acute physiological parameters to achieve more accurate and individualized risk stratification in elderly patients undergoing emergency abdominal surgery.

## Supplementary Information

Supplementary Material 1.

## Data Availability

The datasets generated and/or analyzed during the current study are available from the corresponding author on reasonable request.
