# Supplementary material for: NELA, P-POSSUM, and Paraspinal Muscle Index in risk stratification of elderly patients undergoing emergency laparotomy
Source: BMC Geriatr. 2026 May 11;26:900. doi: 10.1186/s12877-026-07539-y (PMC13330373; doi:10.1186/s12877-026-07539-y)
Supplement: Supplementary file 1 — Supplementary Material 1. [file 12877_2026_7539_MOESM1_ESM.docx]

| **Outcome** | **AUC (95% CI)** | **Sensitivity % (95% CI)** | **Specificity % (95% CI)** | **LR+ (95% CI)** | **LR− (95% CI)** |
| --- | --- | --- | --- | --- | --- |
| Clavien–Dindo ≥3 | 0.760 (0.668–0.852) | 63.3 (48.3–76.6) | 73.4 (60.9–83.7) | 2.38 (1.52–3.71) | 0.50 (0.34–0.73) |
| Clavien–Dindo ≥4 | 0.768 (0.676–0.860) | 64.9 (47.5–79.8) | 68.4 (56.7–78.6) | 2.05 (1.40–3.01) | 0.51 (0.32–0.81) |
| In-hospital mortality | 0.761 (0.659–0.851) | 76.9 (56.4–91.0) | 67.8 (56.9–77.4) | 2.39 (1.66–3.44) | 0.34 (0.17–0.69) |

**Table Legend**

**Supplementary Table S1.** Diagnostic performance of the Prognostic Nutritional Index (PNI) for predicting major complications (Clavien–Dindo ≥3), life-threatening complications (Clavien–Dindo ≥4), and in-hospital mortality. Receiver operating characteristic (ROC) analyses were performed using continuous PNI values. The optimal cut-off value (PNI ≈ 34.5) was determined using the Youden index. Sensitivity, specificity, and likelihood ratios were calculated at this threshold. Confidence intervals (95% CI) were calculated for all diagnostic performance measures.
